# Temporary Parenteral Tacrolimus Requirement due to Unexplained Low Through Levels in a Liver Transplant Patient with Short Bowel Syndrome 

**Published:** 2014-02-01

**Authors:** V. Ince, F. Ozdemir, B. Isik, M. Yilmaz, C. Kayaalp, S. Yilmaz

**Affiliations:** *Inonu University, Liver Transplantation Institute, Malatya, Turkey*

**Keywords:** Tacrolimus, Living donor, Liver transplantation, Short bowel syndrome, immunosuppressive agent.

## Abstract

An adequate level of tacrolimus in serum should be obtained to prevent acute rejection following liver transplantation. Because of good gastrointestinal absorption of oral tacrolimus, adequate trough levels can be achieved even in patients with short bowel syndrome. Rarely, adequate through levels cannot be obtained by oral administration of the drug for several reasons such as inadequate absorption, having a discordant patient, laboratory error, and/or interactions with other drugs and foods. Here, we described a 16-year-old patient who had undergone massive intestinal resection due to mesenteric torsion 5 years previously and required liver transplantation for cryptogenic cirrhosis. Her remnant small bowel length was 90 cm. After a successful living donor liver transplantation, oral tacrolimus administration resulted in inadequate through levels in some parts of the postoperative period. We checked up all the potential reasons but could not identify any cause. An intravenous tacrolimus including immunosuppressive regimen was temporarily required. She maintained adequate blood levels of tacrolimus by parenteral route for a while; thereafter, oral administration resulted in enough blood drug levels. She was discharged with oral tacrolimus therapy. We concluded that very rarely, adequate blood levels of tacrolimus cannot be achieved by oral administration for unexplained reasons. In such cases, temporary administration of parenteral tacrolimus can be life-saving.

## INTRODUCTION

Adequate immunosuppression is essential for graft survival after liver transplantation. Oral tacrolimus is one of the most frequently administered immunosuppressive agents after liver transplantation. Absorption after oral administration of tacrolimus, is normally adequate to provide an appropriate blood level, even in patients with short bowel syndrome [1, 2]. Intravenous tacrolimus therapy after liver transplantation has been attempted only rarely either in a clinical setting or in experimental studies [2-6]. Herein, we present a liver transplant patient with short bowel syndrome for whom we had to switched tacrolimus from oral to intravenous route for a short period of time. Despite enough intestinal length for drug absorption and adequate oral intake, we could not manage normal trough levels of tacrolimus. Short-term parenteral tacrolimus support was used with safety when there was an inability to absorb it per oral. 

## CASE REPORT

A 16-year-old female patient who had been suffering from jaundice and general malaise for three months was diagnosed with subacute hepatic failure and referred to our clinic for liver transplantation. The patient had undergone a massive intestinal resection due to mesenteric torsion five years previously. According to the surgical records, the remnant length of the small intestine was 90 cm. She had diarrhea five times daily post-operatively and required enteral nutrition, but total parenteral nutrition had not been required while at home. Grade 1 hepatic encephalopathy, jaundice, minimal ascites and a midline incision scar were observed on physical examination. On admission, she had an international normalized ratio (INR) of 1.8, serum total bilirubin of 61.6 mg/dL, direct bilirubin of 40.9 mg/dL, albumin of 3.6 g/dL, ammonia of 262 µg/dL, aspartate aminotransferase (AST) of 437 U/L, and alanine aminotransferase (ALT) of 240 U/L. The CHILD score/class of the patient was 10/C; the MELD score was 29. Laboratory tests did not reveal any obvious etiology for the observed findings. The patient was tentatively diagnosed with liver disease associated with hepatic failure. Multidetector computed tomography (MDCT) showed normal hepatic margins, homogeneous hepatic parenchyma, splenomegaly, and minor ascites. During the observation period, total bilirubin levels always measured over 35 mg/dL despite intensive therapies and blood ammonia levels increased to 695 µg/dL, respectively. The hepatic encephalopathy also failed to resolve, so liver transplantation was planned. The patient’s elder brother was prepared as a living donor. 

After the removal of extensive adhesions, the remaining small intestine was measured at 90 cm and the stomach, duodenum, ileocecal valve and colon were intact. The liver had a micronodular cirrhotic appearance. Right lobe living related donor liver transplantation was performed uneventfully. The pathology of the patient’s replaced liver confirmed active cirrhosis with submassive necrosis.

**Figure 1:**
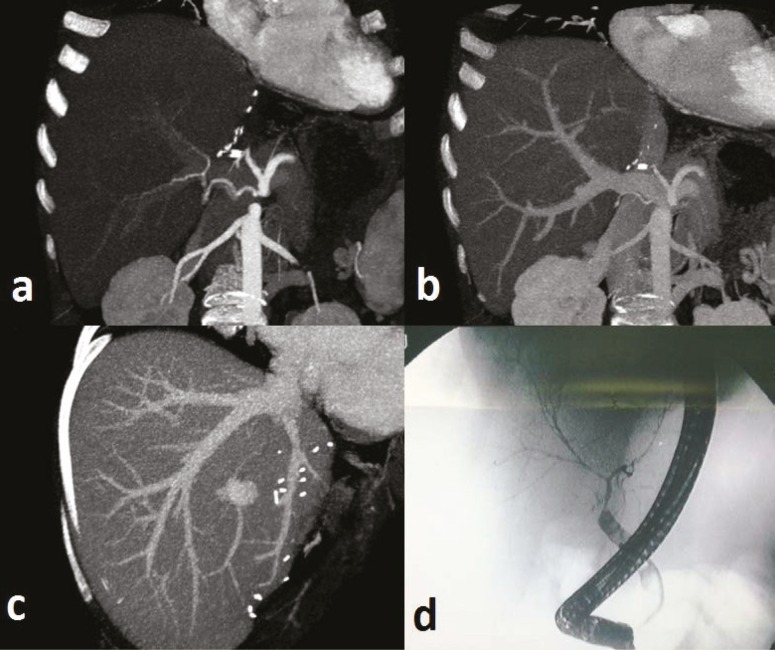
No pathological findings were observed in vascular structures of the liver on MDCT and ERCP. a) Arteria hepatica and brunches; b) Right portal vein; c) Hepatic veins, and d) Bile ducts as normally on ERCP imaging

**Figure 2:**
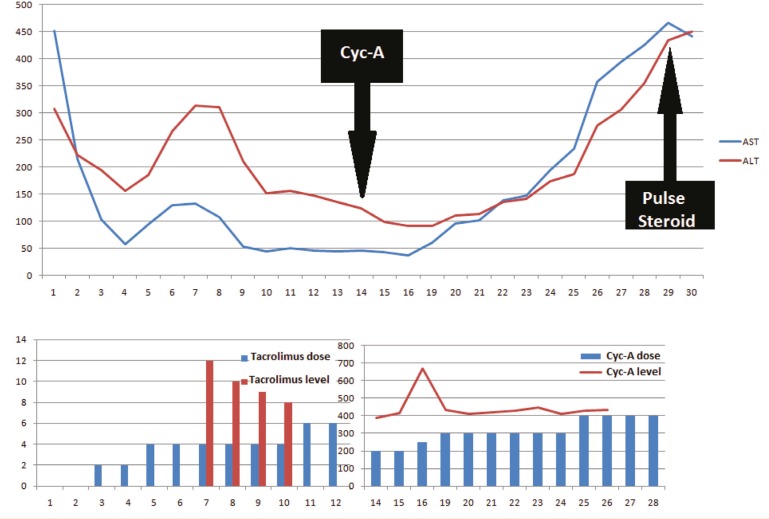
Switch to cyclosporine (Cyc-A). Pulse steroid and re-administration of orally tacrolimus

**Figure 3:**
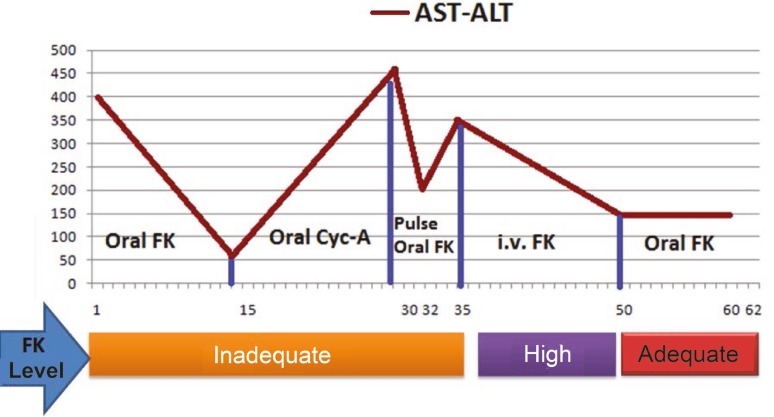
Switch to oral tacrolimus following intravenous tacrolimus

Oral tacrolimus at 2 mg/day, mycophenolate mofetil (MMF) at 1000 mg/day, and intravenous prednisolone at 100 mg/day were given as immunosuppressive therapy post-operatively. The dose of tacrolimus therapy was increased to 6 mg/day and this was adjusted based on changes in liver function tests. Unfortunately, the patient had a seizure while receiving tacrolimus on postoperative day 15, so cyclosporine was administered instead. Cyclosporine concentrations at 2 hour post-dose (C2 level) were regulated at 400 to 500 ng/mL, but liver function remained compromised. Potential pathological disorders of vascular and biliary structures were excluded using MDCT and diagnostic ERCP (Fig 1). The patient had no evidence of infectious disease, and then was diagnosed with acute rejection, which did not respond to increased dose cyclosporine therapy. Pulse steroid therapy was started and immunosuppressive treatment was switched again to tacrolimus (Fig 2). Liver enzymes (AST, ALT) decreased rapidly after initial therapy, but although the tacrolimus dose was 10 mg/day, on the fifth day of pulse therapy, the serum tacrolimus level was still under 5 ng/mL. No neurological complications were observed under re-treatment with tacrolimus. Patient compliance with the medications was confirmed by a nurse. Other drugs and foods that could affect the blood level were checked. Laboratory devices were re-calibrated prior to the tests. Due to inadequate blood levels, despite taking the same dose of tacrolimus (10 mg/kg) for three days, and the increase in serum AST and ALT made us to start to administrate intravenous tacrolimus at a dosage of 2 mg/day at the end of the third day. Appropriate blood levels of tacrolimus were reached easily (15-20 ng/mL) by intravenous way. Then liver enzymes (AST, ALT) decreased. After two weeks of intravenous therapy, oral tacrolimus was re-administered at 4 mg/day, risking neurological complications but obtaining a serum tacrolimus level of 10-15 ng/mL. The patient continued maintenance therapy at 4 mg/day tacrolimus and was discharged in a good health with the same tacrolimus dosage (Fig 3). However, on the sixth post-operative month, the patient presented to our clinic due to fever and malaise. She was treated medically, but ultimately died of severe sepsis and multiorgan failure. 

## DISCUSSION

Tacrolimus is absorbed predominantly by the proximal small intestine. Intestinal metabolites formed after oral administration of tacrolimus are present at the highest concentrations in the duodenum and jejunum, where the drug is mainly absorbed, and decline at the ileum and colon [1, 7]. However, enterocytes in the small intestine express high levels of the CYP3A4 family of metabolizing enzymes in the endoplasmic reticulum, which is responsible for tacrolimus pharmacokinetics [8-10]. CYP3A5 polymorphism affects the metabolism of tacrolimus. These genotype combinations can alter the concentration of tacrolimus in the blood after oral administration even in those with a normal gut [6]. Following small bowel resection, there is a reduced first-pass effect, resulting in increased peak levels of tacrolimus. Thus, the following paradoxical statement that “the shorter the residual small intestine, the higher the oral tacrolimus blood concentration” is true [6, 9]. Even in patients with ultra-short bowel syndrome, low-dose oral tacrolimus can achieve acceptable therapeutic levels after liver transplantation [2, 6]. Therefore, these studies support this paradoxical situation. 

Because tacrolimus is well absorbed in the gastrointestinal tract, and even its low doses can achieve adequate blood levels, why could an adequate blood level not be obtained in our patient? Possible explanations would include inadequate absorption, having a discordant patient, laboratory error, and/or interactions with other drugs and foods. We address each of these below.

Tacrolimus can reach an adequate blood levels even in patients with short bowel syndrome. Although in our case, the stomach, duodenum, and 90 cm of the jejunum and colon were intact, blood levels of tacrolimus were inadequate. We considered a possible polymorphism of CYP3A, and on re-initiation of oral tacrolimus following parenteral administration, adequate blood levels were achieved.

Our patient was completely cooperative with the treatment and took all medications as directed. If she had not taken the medication, it would have been impossible to measure any blood levels of tacrolimus. In this case, the level was measurable, but inadequate, so we know that her compliance was acceptable. Besides, patient compliance with the medications was confirmed one-to-one by a transplant nurse as well. We contacted the laboratory staff, who re-calibrated the devices, after which measurements were repeated. The results were unchanged and there were no unexpected results seen with any other liver transplant patients. We researched possible interactions between tacrolimus and other drugs/foods, but found nothing significant. 

Therefore, we were unable to discover the reason behind inadequate tacrolimus levels in the serum, and intravenous tacrolimus did obtain adequate levels temporarily. Furthermore, the medication had to be stopped for a few days because the blood level reached over 25 ng/mL. Oral tacrolimus therapy at 4 mg/day was initiated after two weeks of intravenous therapy, which the patient tolerated well and an adequate level was reached.

Very rarely, adequate blood levels of tacrolimus cannot be achieved by oral administration for unexplained reasons. In such cases, intravenous tacrolimus can be used safely.
